# Urinary N-acetyl-β-D glucosaminidase as a surrogate marker for renal function in autosomal dominant polycystic kidney disease: 1 year prospective cohort study

**DOI:** 10.1186/1471-2369-13-93

**Published:** 2012-08-30

**Authors:** Hayne Cho Park, Jin Ho Hwang, Ah-Young Kang, Han Ro, Myung-Gyu Kim, Jung Nam An, Ji In Park, Seung Hyup Kim, Jaeseok Yang, Yun Kyu Oh, Kook-Hwan Oh, Jung Woo Noh, Hae Il Cheong, Young-Hwan Hwang, Curie Ahn

**Affiliations:** 1 Department of Internal Medicine, Seoul National University College of Medicine, 101 Daehak-Ro Jongno-Gu, Seoul, 110-744, South Korea; 2 Research Center for Rare Diseases, Seoul National University Hospital, Seoul, South Korea; 3 Transplantation Center, Seoul National University Hospital, Seoul, South Korea; 4 Department of Internal Medicine, Gacheon University Gil Hospital, Incheon, South Korea; 5 Department of Radiology, Seoul National University College of Medicine, Seoul, South Korea; 6 Department of Internal Medicine, Hallym University College of Medicine, Seoul, South Korea; 7 Department of Pediatrics and Adolescent Medicine, Seoul National University College of Medicine, Seoul, South Korea; 8 Kidney Research Institute, Seoul National University Hospital, Seoul, South Korea; 9 Department of Internal Medicine, Eulji General Hospital, Eulji University 280-1 Hagye 1-dong, Nowon-gu, Seoul, 139-711, Korea

**Keywords:** Autosomal dominant polycystic kidney disease, Biomarkers, Renal function

## Abstract

**Background:**

Renal failure is one of the most serious complications associated with autosomal dominant polycystic kidney disease (ADPKD). To date, early markers have failed to predict renal function deterioration at the early stages. This 1-year prospective study evaluated N-acetyl-β-D-glucosaminidase (NAG) as a new surrogate marker for renal function in ADPKD.

**Methods:**

A total of 270 patients were enrolled in the study, and we measured urinary NAG, β2-microglobulin, neutrophil gelatinase-associated lipocalin (NGAL) and kidney injury molecule-1 (KIM-1) prospectively for 1 year to compare their predictive values for renal function.

**Results:**

Baseline urinary NAG/Cr was negatively correlated with estimated glomerular filtration rate (GFR) (*r*^*2*^ = 0.153, *P* < 0.001) and positively correlated with total kidney volume (TKV) (*r*^*2*^ = 0.113, *P* < 0.001). Among other biomarkers, urinary NAG/Cr better discriminated patients with decreased renal function from those with conserved renal function, showing the largest area under the curve (AUC 0.794). Immunohistochemical study revealed strong staining along the cyst-lining epithelial cells as well as the nearby compressed tubular epithelial cells. However, both single and repeated measurements of urinary NAG/Cr failed to predict renal function decline in 1 year.

**Conclusions:**

Urinary NAG/Cr may be a useful surrogate marker for renal function in ADPKD patients.

## Background

Autosomal dominant polycystic kidney disease (ADPKD) is the most common hereditary renal disease. In ADPKD, renal function deteriorates progressively as cysts replace the renal parenchyma. However, cysts grow significantly earlier than a decline in renal function is observed [1]. Therefore, serum creatinine (sCr) and the glomerular filtration rate (GFR) cannot properly reflect disease progression in ADPKD patients. Chapman et al. demonstrated that total kidney volume (TKV) is a reliable surrogate marker for renal function in the early stage of ADPKD [2]. However, imaging tools are impractical because they are time-consuming and expensive to perform repeatedly. Therefore, there has been a growing need for novel biomarkers that are easy, fast, and economical to assess.

The N-acetyl-β-D-glucosaminidase (NAG) is a 130-kD glycolytic lysosomal enzyme widely distributed among different types of tissue cells [3]. In kidneys, NAG is mainly distributed in the lysosome of the proximal convoluted tubules [4]. In previous studies, NAG has been suggested to be elevated in various renal diseases and is considered to be an early sensitive indicator of acute renal tubular injury [5]. However, NAG has not been thoroughly investigated in ADPKD patients. Therefore, this 12-month prospective study was performed to evaluate NAG as a potential surrogate biomarker for renal function in ADPKD.

## Methods

### Study population

From 1984 to 2011, a total of 456 ADPKD patients were registered at the Seoul National University Hospital ADPKD clinic. ADPKD was diagnosed according to the Unified Criteria for Ultrasonographic Diagnosis of ADPKD proposed by Pei et al. [6]. Of the 456 ADPKD patients, 270 were enrolled in this study after providing informed consent. These patients were between 18 and 60 years in age and had estimated GFRs over 30 mL/min/1.73 m^2^. The following patients were excluded: those with acute illnesses, infections or acute kidney injury within the past 3 months; patients with chronic illnesses such as connective tissue disease, chronic hepatitis, cancer, rheumatoid disease, or thyroid disease; those with history of organ transplantation or nephrectomy; those who have been maintained on renal replacement therapy; pregnant women; and patients who either refused to enroll in the study or to undergo imaging due to contrast nephrotoxicity.

### Data collection

Upon enrollment, information on age, gender, age at diagnosis, family history (cerebro-vascular accident and end-stage renal disease), co-morbidities (diabetes mellitus, coronary artery disease, heart failure, and peripheral vascular disease), renal and extra-renal complications, and duration of hypertension were collected. Follow-up data were collected every 6 months. On each visit, systolic and diastolic blood pressures were measured. The number and types of anti-hypertensive medications were also reviewed. Laboratory parameters were collected every 6 months. In addition, we reviewed the past medical record to collect information on sCr and GFR at the initial visit. The sCr was measured using the Jaffe method. The GFR was calculated using both the isotope dilution mass spectrometry (IDMS)-traceable Modification of Diet in Renal Disease (MDRD) equation and CKD-EPI (Chronic Kidney Disease Epidemiology) equation [7,8]. The mean follow-up duration after the first visit was 11.0 ± 1.19 months.

### Measurement of TKV

Instead of using MRI, we have evaluated TKV from recent contrast-enhanced CT images. All patients underwent three-dimensional contrast-enhanced CT of the kidney and bladder using a multi-detector CT scanner. The CT examination was performed using a spiral technique with 3- to 5-mm thickness. Renal volume was measured using the modified ellipsoid method [9]. The TKV was defined as the sum of the left and right renal volumes.

### Measurement of urinary biomarkers

The activity of NAG was measured with a spectrophotometric assay under a 340-nm wavelength using a TBA 200 FR biochemical analyzer (Toshiba, Tokyo, Japan) [10]. To evaluate whether urinary NAG is the best marker of renal function and kidney enlargement, we also measured other urinary biomarkers such as NGAL, KIM-1, and β2-microglobulin. To measure urinary NGAL and KIM-1, we separately collected 10 mL of random spot urine samples in polypropylene bottles containing 1 mL of 10 mM Tris buffer. Then, a 1-mL aliquot of the sample was centrifuged at 3,000 x g for 10 minutes and stored at −70°C until the measurement. Urinary NGAL and KIM-1 levels were measured using an enzyme-linked immunosorbent assay (BioPorto Diagnostics, Denmark; R & D Systems, China). For NGAL measurements, 1:100 diluted urine was used. The urinary excretion level of β2-microglobulin was measured using a β2-microglobulin radioimmunoassay kit by a previously reported method (Beckman Coulter, Prague, Czech Republic) [11]. To compensate for the production of concentrated or dilute urine samples, the values of NAG and β2-microglobulin were expressed based on urinary creatinine content.

### Immunohistochemistry of NAG

Formalin-fixed, paraffin-embedded polycystic and control kidney blocks were used for immunohistochemistry. The procedures for the use of the human kidney specimens were approved by Seoul National University Hospital Institutional Review Board. The ADPKD kidney specimen was obtained from a 47-year-old woman who underwent right nephrectomy for renal cell carcinoma. Her sCr level and eGFR prior to operation were 0.99 mg/dL and 60.12 mL/min/1.73 m^2^, respectively. The control kidney used in this study was from a 70-year-old man who underwent left nephrectomy for renal cell carcinoma. His sCr level and eGFR prior to operation were 1.0 mg/dL and 58.3 mL/min/1.73 m^2^, respectively.

Four-micron frozen tissue sections were prepared for immunohistochemistry as described previously [12]. Primary antibody (Anti-MGEA5 rabbit monoclonal antibody, Epitomics, Burlingame, CA) was added at a dilution of 1:450, followed by the addition of the secondary antibody (ZytoChem Plus HRP One-step Polymer anti-Mouse/Rabbit/Rat, Zytomed System, Berlin, Germany). The DAB substrate was given to reveal purple or brown color under light microscopy. The sections were counterstained with hematoxylin (Surgipath Medical Industry, Inc., IL, USA), viewed under an Olympus microscope BX51, and photographed.

### Statistical analyses

Analyses were performed using SPSS, version 19.0 (SPSS Inc., http://www.spss.com) and R version 2.13.2 (http://www.r-project.org). To investigate whether urinary biomarkers were correlated with either estimated GFR or TKV, linear regression analyses were performed. We also analyzed the reference test as dichotomous variables to calculate receiver operating characteristic (ROC) curves. To adjust for potential confounders, multiple regression analysis was performed. The stepwise selection method was used to define independent factors. Age, gender, hypertension, urinary NAG/Cr, and the presence of albuminuria were included in the final model.

This study was approved by the Institutional Review Board of Seoul National University Hospital (H-0901-046-269). Informed consent was obtained from the subjects in accordance with the Declaration of Helsinki.

## Results

### Clinical characteristics of participants

The mean age of the patients was 43.1 ± 9.6 years, and they were followed up in the ADPKD outpatient clinic for a median of 60 (18–87) months at the time of enrollment (Table 1). Hypertension was noted in 208 patients (77.0%), and the mean systolic and diastolic blood pressures were 130.2 ± 11.4 mmHg and 81.2 ± 8.3 mmHg, respectively. The baseline sCr level was 0.9 ± 0.3 mg/dL and the estimated GFR was 85.0 ± 24.7 mL/min/1.73 m^2^ (min. 30.0, max. 142.0) using IDMS-traceable MDRD equation and 91.9 ± 23.3 mL/min/1.73 m^2^ using CKD-EPI equation (min. 32.0, max. 134.0), respectively. The TKV measured upon enrollment was 1014 mL (min. 211, max. 5324). The chronic kidney disease (CKD) stage was defined according to the estimated GFR as previously described [13]. The proportion of patients with CKD stages II ~ IV increased over time, whereas the proportion of patients with CKD stage I slightly decreased over the same time period (See Additional file 1).

**Table 1:** Clinical characteristics of participants at enrollment

**Parameters**	**Patients (N = 270)**
Age (yr)	43.1 ± 9.6
Male	126 (46.7%)
Age at diagnosis (yr)	34.9 ± 9.5
Hypertension	208 (77.0%)
Duration of hypertension (yr)	7 (4–11)
Number of BP medication	2 (Min. 1, Max. 4)
Mean systolic/diastolic BP (mmHg)	130.2 ± 11.4/81.2 ± 8.3
Family history of ESRD	112 (41.5%)
Total kidney volume (mL)	1014 (Min 211, Max. 5324)
Hemoglobin (g/dL)	13.5 ± 1.53
Creatinine (mg/dL)	0.9 ± 0.3
IDMS-MDRD GFR (mL/min/1.73 m^2^)	85.0 ± 24.7
CKD-EPI GFR (mL/min/1.73 m^2^)	91.9 ± 23.3
Random urine microalbumin-to-creatinine ratio (mg/g)	51.4 ± 105.8

Data are expressed as numbers (percentages), mean ± SD, or median (Min., Max.). BP, blood pressure; CVA, cerebrovascular accident; ESRD, end-stage renal disease.

### Correlation between TKV measured by modified ellipsoid method and estimated GFR

Because we measured TKV by modified ellipsoid method using CT images, we analyzed the association between the measured TKV and estimated GFR before performing other analyses. A total of 270 cases of CT images were used to estimate TKVs. The TKV was log-transformed prior to any analysis because it did not follow a normal distribution. The TKV measured using CT images was negatively correlated with the estimated GFR (*r*^*2*^ = 0.334, *P* < 0.001) (See Additional file 2).

### Initial urinary NAG/Cr value is well correlated with the subsequent urinary NAG/Cr measurements

**Figure 1:**
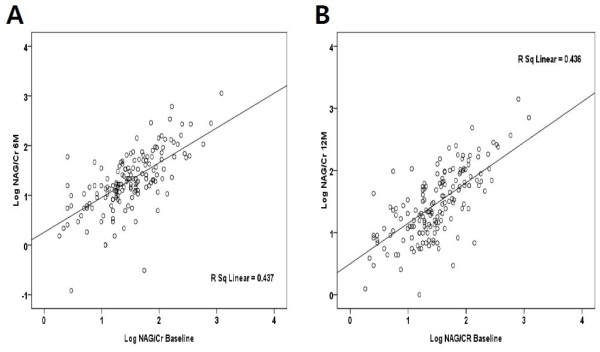
**The association of urinary NAG/Cr levels from different time points.** A linear regression analyses showed that the baseline urinary NAG/Cr level was in a good correlation with subsequent urinary NAG/Cr measurements at 6 months and 12 months (*r*^*2*^ = 0.437 and 0.436, *P* < 0.001).

To evaluate the stability of urinary NAG/Cr measurement in each individual over time, we performed a linear regression analysis between initial urinary NAG/Cr value and subsequent measurements. The scatter plot demonstrated a good correlation between initial urinary NAG/Cr value with follow-up measurement at 6 months and 12 months (*r*^*2*^ = 0.437 and 0.436, *P* < 0.001) (Figure 1).

### Urinary NAG/Cr represents current estimated GFR and TKV

**Figure 2:**
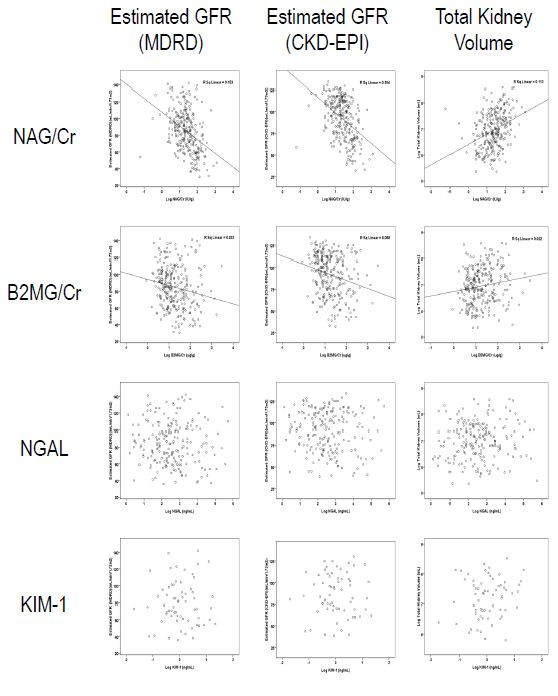
**Linear regression analysis between each urinary biomarker and the IDMS-traceable MDRD GFR (left column), CKD-EPI GFR (middle column), and TKV (right column).** Biomarker concentrations and TKVs were log-transformed to fulfill the requirement of a normal distribution of the residuals. Urinary NAG levels showed a better association with the IDMS-traceable MDRD GFR (*r*^*2*^ = 0.153), CKD-EPI GFR (*r*^*2*^ = 0.194), and TKV (*r*^*2*^ = 0.113) compared with other urinary biomarkers (*P* < 0.001). NAG (A); β2-microglobulin (B); NGAL (C); KIM-1 (D). sCr, serum creatinine; GFR, glomerular filtration rate; KIM-1, kidney injury molecule-1; NAG, N-acetyl-β-D-glucosaminidase; NGAL, neutrophil gelatinase-associated lipocalin; TKV, total kidney volume.

A linear regression analysis was performed between the estimated GFR and urinary biomarkers, including NAG/Cr, β2-microglobulin/Cr, NGAL and KIM-1 (Figure 2). Urinary NAG and β2-microglobulin levels were measured in 270 samples, NGAL levels were measured in 197 samples, and KIM-1 levels were measured in 64 samples. The age (43.4 ± 9.5 vs. 42.2 ± 9.9), gender (M/F = 95/102 vs. 31/42), and ACR (48.7 ± 88.3 vs. 58.3 ± 141.5) did not vary significantly between those with and those without NGAL measurements (*P* > 0.05). Among 270 patients with both NAG and β2-microglobulin measurements, NAG/Cr and β2-microglobulin/Cr were negatively correlated with the IDMS-traceable MDRD GFR (*r*^*2*^ = 0.153 vs. 0.033, *P* < 0.001). The estimated GFR calculated by using CKD-EPI equation showed the similar correlation with biomarkers (*r*^*2*^ = 0.194, *P* < 0.001). Urinary NAG/Cr also showed a positive correlation with TKV (*r*^*2*^ = 0.113, *P* < 0.001). Although urinary β2-microglobulin/Cr exhibited a statistically significant correlation with the estimated GFR, the Pearson’s correlation coefficient was smaller than that of urinary NAG/Cr. In contrast, both NGAL and KIM-1 displayed no correlation with any of the aforementioned parameters.

When we compared urinary NAG/Cr among patients in different CKD stages according to IDMS-traceable MDRD equation, the patients in CKD stage 3 (n = 116) showed higher urinary NAG/Cr level compared to those in CKD stage 1 (n = 109) or 2 (n = 45)(7.06 ± 3.9 vs. 3.7 ± 2.1 or 4.67 ± 2.5, *P* < 0.001)(See Additional file 3). When we divided CKD stages using CKD-EPI equation, the results were similar to that from IDMS-traceable MDRD equation (data not shown).

**Figure 3:**
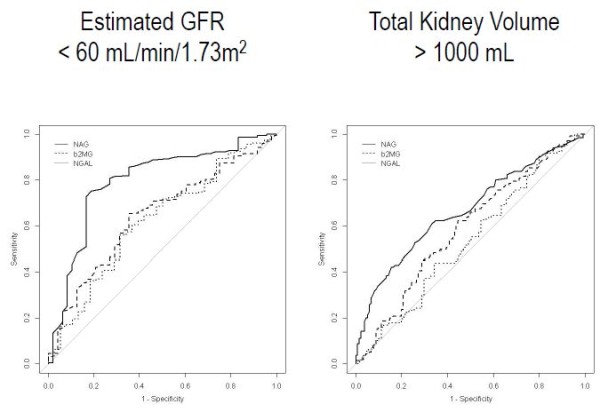
**Receiver operating characteristic curve analysis with urinary biomarkers for estimated GFR < 60 mL/min/1.73 m**^**2**^**(A) and TKV > 1000 mL (B).** Urinary NAG/Cr is a better marker for discriminating patients with decreased estimated GFRs (< 60 mL/min/1.73 m^2^) from those with conserved renal function, showing the largest AUC (0.794). In addition, urinary NAG/Cr can best discriminate patients with enlarged TKV (>1000 mL), showing an AUC of 0.637.

To determine whether urinary NAG/Cr is a better indicator of renal functional and structural markers than urinary β2-microglobulin and NGAL, we performed a ROC curve analysis. At first, urinary biomarker concentrations were compared with the estimated GFR (the reference procedures). Data from 197 baseline samples in which NAG, β2-microglobulin and NGAL were measured were included in the analysis. The sensitivity and specificity were determined, and the area under the curve (AUC) was calculated for the urinary NAG, β2-microglobulin and NGAL levels. Among the other urinary biomarkers, urinary NAG/Cr was a better indicator for discriminating patients with decreased estimated GFRs (< 60 mL/min/1.73 m^2^) from those with conserved renal function, showing the largest AUC (0.794) (Figure 3). The sensitivity and specificity of urinary NAG/Cr was 75.2% and 81.2%, respectively, with a cut-off value of 4.95 IU/g. The urinary NAG/Cr was also a better indicator for discriminating patients with enlarged kidneys (>1000 mL) from those with smaller kidneys, showing the largest AUC (0.637).

### Urinary NAG/Cr is an independent variable of estimated GFR

In linear regression analyses (Table 2), age, years from diagnosis, hypertension, degree of albuminuria, and urinary NAG/Cr were associated with the estimated GFR (*P* < 0.05). In multiple regression analyses, urinary NAG/Cr remained as a significant risk factor after adjusting for other variables (unstandardized coefficient, 95% CI; -4.5, -8.6 to −0.5, *P* = 0.03).

**Table 2:** Linear regression analysis for risk factors for decreased estimated glomerular filtration rate

**Parameters**	**Univariable**		**Multivariable**	
	**B (95 % CI)**	***P*value**	**B (95 % CI)**	***P*value**
Age (yr)	−1.4 (−1.6 to −1.1)	< 0.001	−1.1 (−1.3 to −0.8)	< 0.001
Gender	0.7 (−4.7 to 6.2)	0.797	5.1 (0.7 to 9.5)	0.022
Years from diagnosis	−1.3 (−1.7 to −0.9)	< 0.001	-	-
Hypertension	−23.4 (−29.2 to −17.6)	< 0.001	−8.5 (−14.0 to −3.0)	0.003
Albumin/Cr (mg/g)	−9.3 (−11.7 to −7.0)	< 0.001	−5.6 (−7.6 to −3.6)	< 0.001
NAG/Cr (IU/g)	−16.5 (−20.7 to −12.3)	< 0.001	−4.5 (−8.6 to −0.5)	0.03

Data were adjusted for age, years from diagnosis, hypertension and albuminuria. Albumin/Cr and NAG/Cr were log-transformed to fulfill the requirement of normal distribution of residuals. Cr, creatinine; NAG, N-Acetyl-β-D-glucosaminidase.

### Single measurement of urinary NAG/Cr could not predict renal function decline in 1 year

**Figure 4:**
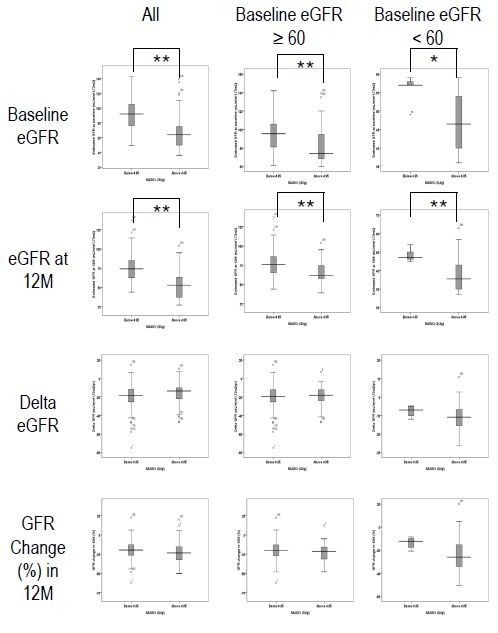
**Single measurements of NAG/Cr could not predict renal function deterioration in 1 year.** As a whole, the patients with a high baseline urinary NAG/Cr (≥ 4.95 IU/g) demonstrated lower estimated GFRs at baseline and at 12 months (54.8 ± 19.3 vs. 75.3 ± 18.7, *P* < 0.001). However, annual decline of estimated GFRs were not different between high and low NAG/Cr groups. Subgroup analyses in the patients with baseline estimated GFR ≥ 60 and < 60 mL/min/1.73 m2 showed the similar results. Baseline estimated GFR (A); estimated GFR at 12 months (B); annual change in GFR over 12 months (C); percentage decrement in 12 months (D).

To elucidate whether a single measurement of urinary NAG/Cr predicts ADPKD progression, we performed an independent *t*-test to compare the mean values of 1) baseline estimated GFR, 2) estimated GFR at 12 months, 3) absolute GFR change in 12 months, and 4) percentage GFR change in 12 months between low and high baseline urinary NAG/Cr groups (Figure 4). Low (< 4.95 IU/g) and high (≥ 4.95 IU/g) NAG/Cr were defined based on the cut-off value used in the previous ROC curve analyses. Among the 164 patients with available follow-up samples at 12 months, 61 patients were included in the high NAG/Cr group (7.89 ± 3.19 IU/g) and 103 patients were included in the low NAG/Cr group (3.27 ± 1.1 IU/g). As the result, the high urinary NAG/Cr group showed lower estimated GFR at baseline (68.0 ± 24.6 vs. 92.6 ± 20.3 mL/min/1.73 m^2^, *P* < 0.001) and at 12 months (53.3 ± 18.7 vs. 74.9 ± 18.0 mL/min/1.73 m^2^, *P* < 0.001). However, the annual change of estimated GFR was not significantly different in either absolute value (−16.1 ± 11.6 vs. -19.6 ± 13.5 mL/min/1.73 m^2^/yr, *P* = 0.08) or percentage change (−22.9% vs. -20.7%, *P* = 0.3) between high and low NAG/Cr groups. Since the baseline estimated GFR may affect the subsequent GFR decline rate, we divided the patients into two groups: estimated GFR ≥ 60 and < 60 mL/min/1.73 m^2^. When we performed subgroup analyses, however, the annual change of estimated GFR was not different between high and low NAG/Cr groups. The results of our analysis indicated that single measurement of urinary NAG/Cr may not predict the renal function decline in 1 year.

### Repeated measurements of urinary NAG/Cr may not indicate faster GFR decline in 1 year

Finally, we performed ANOVA to determine whether persistently high level of urinary NAG/Cr is an indicator of renal function deterioration (See Additional file 4). A total of 161 patients with available baseline and 6- and 12-month data were included in the analysis. Patients were divided into three groups based on serial measurements of the urinary NAG/Cr over a 12-month period: persistently low urinary NAG/Cr (<4.95 IU/g) (Group L-L, n = 78), variable urinary NAG/Cr (Group V, n = 54) and persistently high urinary NAG/Cr (≥ 4.95 IU/g) (Group H-H, n = 29). The estimated GFR at baseline was significantly lower in the Group H-H compared to the Group V or the Group L-L (64.5 ± 23.3 vs. 77.6 ± 23.6 vs. 94.2 ± 19.8 mL/min/1.73 m^2^, *P* < 0.001). In addition, the estimated GFR at 12 month was significantly lower in the Group H-H compared to the Group V or the Group L-L (50.3 ± 18.5 vs. 63.0 ± 19.5 vs. 75.7 ± 18.0 mL/min/1.73 m^2^, *P* < 0.001). However, the percentage decrement of estimated GFR was not different between groups (Group H-H vs. Group V vs. Group L-L, -23.0% vs. -20.8% vs. -21.2%, *P* = 0.77). The results of our analysis indicated that repeated measurement of urinary NAG/Cr may not predict the renal function decline in 1 year.

### NAG is produced by both cyst lining epithelial cells and compressed tubular epithelial cells in ADPKD

**Figure 5:**
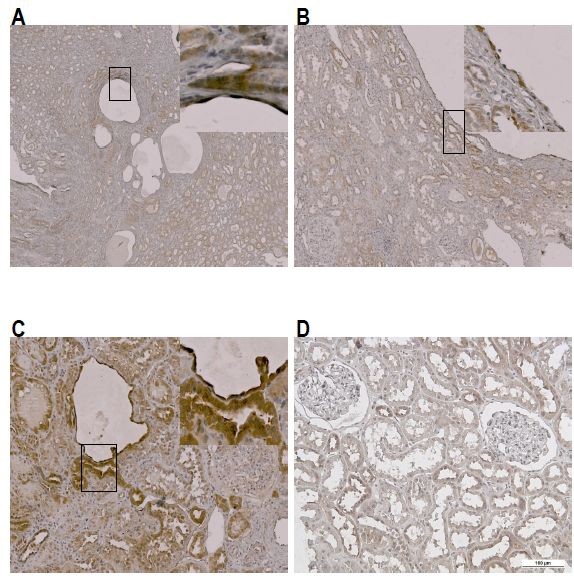
**Immunohistochemisty staining of NAG.** The NAG expression in ADPKD kidney. **(A)** ~ **(C)** Anti-NAG antibody was strongly stained along the cystic epithelial lining cells and adjacent compressed renal tubular epithelial cells. **(D)** NAG expression in normal kidney.

To elucidate the origin of NAG production in ADPKD kidneys, we performed immunohistochemical stains using an anti-MGEA5 antibody (Epitomics, Burlingame, CA) (Figure 5). Compared with normal kidney tissue, NAG was strongly stained upon the cyst-lining epithelial cells and the proximal tubular epithelial cells. Moreover, the proximal tubular epithelial cells that were compressed by nearby cysts stained more strongly than non-compressed normal tubules.

## Discussion

Our study suggests that the urinary NAG/Cr may represent a useful functional and structural marker in ADPKD patients. A single measurement of urinary NAG was well correlated with concurrent estimated GFR and TKV. In addition, when compared with other urinary biomarkers, urinary NAG levels showed a better association with renal functional and structural markers.

In fact, NAG has several advantages for use as a novel biomarker. First, it is relatively stable with little diurnal variation [14]. Second, it is a sensitive marker for tubular damage. Subtle alterations in the epithelial cells in the brush border of the proximal tubules result in shedding of NAG into the urine, and the amount of shed enzyme can be directly correlated to tubular injury [3]. Third, NAG can be easily measured and quantified by spectrophotometric assay [3]. However, NAG, a sensitive renal tubular injury marker, has not been thoroughly investigated in ADPKD patients.

Meanwhile, several factors have been suggested to contribute to hypertension and renal failure in ADPKD. First, Chapman et al. reported that decreased renal blood flow (or increased vascular resistance) is an independent predictor of renal function decline [2]. Another study demonstrated morphological evidence of vascular malformation using angiograms. The authors suggested that neo-vascularization, which may be caused by cystic compression of adjacent tissue, is a key factor for renal progression [15]. Some investigators have suggested that intrarenal activation of the renin-angiotensin system, which is likely the result of renal ischemia, may play an important role in disease progression [16,17]. Eckardt et al. demonstrated increased erythropoietin formation in interstitial cells juxtaposed with proximal tubular cysts in ADPKD patients [18]. Results from these studies point to the importance of ischemic insult and subsequent vascular remodeling in the disease progression of ADPKD.

When we performed immunohistochemical staining of NAG, staining was strongest along the cyst-lining epithelial cells and the proximal tubular epithelial cells. In particular, the cells that were compressed by nearby cysts were more strongly stained than normal control cells, suggesting that ischemic insult by cyst growth to the adjacent tissue may be the key factor in the production of NAG. Moreover, most of the enlarged cysts do not have any connection with the original tubules, and therefore NAG production from the cyst-lining epithelial cells is less likely to contribute to the urinary NAG level. Increased urinary NAG may be better explained by NAG production from the nearby compressed tubules. Therefore, urinary NAG may represent ongoing ischemic insult in ADPKD patients.

In our study, urinary NAG/Cr was correlated with current TKV. We measured TKV by modified ellipsoid method from CT images. Most previous studies measured TKV by computer-based volumetry using magnetic resonance imaging (MRI). We used CT images in our patients because it is a more useful tool to diagnose renal stones or obstructive uropathy and because it is less expensive than MRI. The modified ellipsoid method was developed to measure kidney volumes from ultrasonographic images. It underestimates TKV when compared with MRI volumetry [19]. However, it is easy, economical, and useful to evaluate renal enlargement in serial measurements. Although the modified ellipsoid method has not been used for CT images, our results are comparable to that from the CRISP study (R = −0.54, *P* < 0.001 vs. R = −0.37, *P* < 0.001). The correlation between urinary NAG/Cr and TKV was weaker than that with the estimated GFR. The reasons for this remain to be further elucidated, but one possible explanation is that cysts are small in the early stages and therefore inadequate to produce ischemic insults to the adjacent tissue.

Previously, Meijer et al. investigated the role of NAG in their cross-sectional study with 102 ADPKD patients and 102 age- and sex-matched control subjects [20]. The authors concluded that NGAL was the most useful marker for ADPKD disease progression. However, their results should be interpreted cautiously because they included relatively small number of patients and mainly Caucasian. In addition, because we included patients with relatively conserved renal function (IDMS-traceable MDRD GFR 85.0 ± 24.7 vs. 68 ± 27 mL/min/1.73 m^2^) and smaller TKVs (1014 [211–5324] vs. 1500 [900–2200] mL) than Meijer’s population, the urinary NAG/Cr may be the better marker to represent early disease progression in ADPKD patients. Moreover, we demonstrated the possible link between cyst growth and pronounced NAG production from compressed tubules. Therefore, urinary NAG/Cr may be more useful surrogate marker for renal function that shows underlying pathogenesis of ADPKD disease progression.

Unfortunately, our study failed to prove the role of urinary NAG/Cr in the prediction of renal function deterioration in 1 year. In our study, both single and repeated measurements of urinary NAG/Cr were not able to predict a decline in the estimated GFR. However, the patients with higher baseline NAG/Cr (≥ 4.95 IU/g) showed relatively higher annual percentage decrement in estimated GFR (−22.9% vs. -20.7%, P = 0.3). In addition, the persistently high NAG/Cr (Group H-H ≥ 4.95 IU/g) group showed higher annual percentage decrement in estimated GFR compared to the other groups (Group H-H vs. Group V vs. Group L-L, -23.0% vs. -20.8% vs. -21.2%, *P* = 0.77). Our findings are in the same context with the results from the recent 3-year prospective study demonstrated by CR Parikh et al. [21]. They showed both urinary Il-18 and NGAL levels were elevated in ADPKD but did not correlate with worsening in kidney function or increase in TKV. Since the natural course of ADPKD is indolent in the early stage, 1 year of follow-up seems too short to evaluate the renal function decline in our cohort. Moreover, the effect of baseline estimated GFR is too strong to see the independent effect of urinary NAG/Cr upon the subsequent renal function decline. Therefore, we assume that longer period of follow-up time is necessary to evaluate the rate of renal function decline.

This is the first 1-year prospective study demonstrating the usefulness of urinary NAG/Cr as a surrogate marker for renal function in ADPKD. To the best of our knowledge, our study evaluated the largest ADPKD cohort to date for a urinary biomarker study. However, our study has a few limitations. This is a single-center study that included only Korean patients. Second, we excluded patients in advanced CKD stages. Therefore, further large-scale studies should be conducted to extend our results to other populations. Third, we measured TKV using a modified ellipsoid method from CT images. Although our results are comparable with CRISP results, validation is needed to using this method in CT volumetry. Finally, long-term follow-up data should be analyzed later to determine whether urinary NAG is useful in the prediction of renal function decline.

## Conclusions

In conclusion, we found that high urinary NAG/Cr was associated with lower estimated GFR and larger TKV. Further long-term studies should reveal the usefulness of urinary NAG/Cr in the prediction of faster renal function deterioration.

## Abbreviations

ACR: Albumin-to-creatinine ratio; ADPKD: Autosomal dominant polycystic kidney disease; ANOVA: Analysis of variance; AUC: Area under the curve; CKD: Chronic kidney disease; CKD-EPI: Chronic Kidney Disease Epidemiology; CT: Computed tomography; GFR: Glomerular filtration rate; IDMS: Isotope dilution mass spectrometry; KIM-1: Kidney injury molecule-1; MDRD: Modification of Diet in Renal Disease; MRI: Magnetic resonance imaging; NAG: N-acetyl-β-D-glucosaminidase; NGAL: Neutrophil gelatinase-associated lipocalin; ROC: Receiver operating characteristic; sCr: Serum creatinine; TKV: Total kidney volume.

## Competing interests

The authors declare that they have no competing interests.

## Authors’ contributions

HP participated in the design of the study and biomarker measurement and drafted the manuscript. JH, JA, JP helped to collect samples and demographic information. AK carried out immunohistochemical staining. HR, MK, JY participated in the statistical analyses. SK carried out kidney volume measurement. YO, KO, JN, HC enrolled the patients at the clinic and collected samples and clinical information. YH conceived of the study, and participated in its design and coordination and helped to draft the manuscript. CA participated in the design of the study, interpretation of data and drafting and revising the manuscript. All authors read and approved the final manuscript.

## Pre-publication history

The pre-publication history for this paper can be accessed here:

http://www.biomedcentral.com/1471-2369/13/93/prepub

## Supplementary Material

Additional file 1 **Patient distribution according to chronic kidney disease stages.** The proportion of advanced CKD stages (IIIB and IV) increased as time passed. Click here for file

Additional file 2 **Linear regression analysis between the estimated glomerular filtration rate and total kidney volume.** The TKV was negatively correlated with the estimated GFR (*r*^*2*^ = 0.334, *P* < 0.001). Click here for file

Additional file 3 **Urinary NAG/Cr according to chronic kidney disease stages.** Patients in chronic kidney disease (CKD) stage III (estimated GFR < 60 mL/min/1.73 m^2^) showed significantly higher urinary NAG/Cr compared with CKD stage I or II (6.48 ± 3.79 vs. 3.7 ± 2.13 vs. 4.67 ± 2.47, *P* < 0.001). Click here for file

Additional file 4 **Repeated measurements of NAG/Cr could not predict renal function deterioration in 1 year.** The patients were divided into three groups according to serial measurements of urinary NAG/Cr: persistently low NAG/Cr (<4.95 IU/g) (Group L-L), variable NAG/Cr (Group V), and persistently high NAG/Cr (≥4.95 IU/g) (Group H-H). Group H-H showed lower estimated GFRs at baseline (64.5 ± 23.3 mL/min/1.73 m^2^) and lower estimated GFR at 12 month (50.3 ± 18.5 mL/min/1.73 m^2^) compared to other groups (*P* < 0.001). Although statistically insignificant, the percentage decrement of estimated GFR over 12 months were greater in Group H-H compared to the other groups (Group H-H vs. Group V vs. Group L-L, -23.0% vs. -20.8% vs. -21.2%, *P* = 0.77). Click here for file
