# Supplementary figures and images for: Urinary N-acetyl-β-D glucosaminidase as a surrogate marker for renal function in autosomal dominant polycystic kidney disease: 1 year prospective cohort study
Source: BMC Nephrol. 2012 Aug 30;13:93. doi: 10.1186/1471-2369-13-93 (PMC3465238; doi:10.1186/1471-2369-13-93)

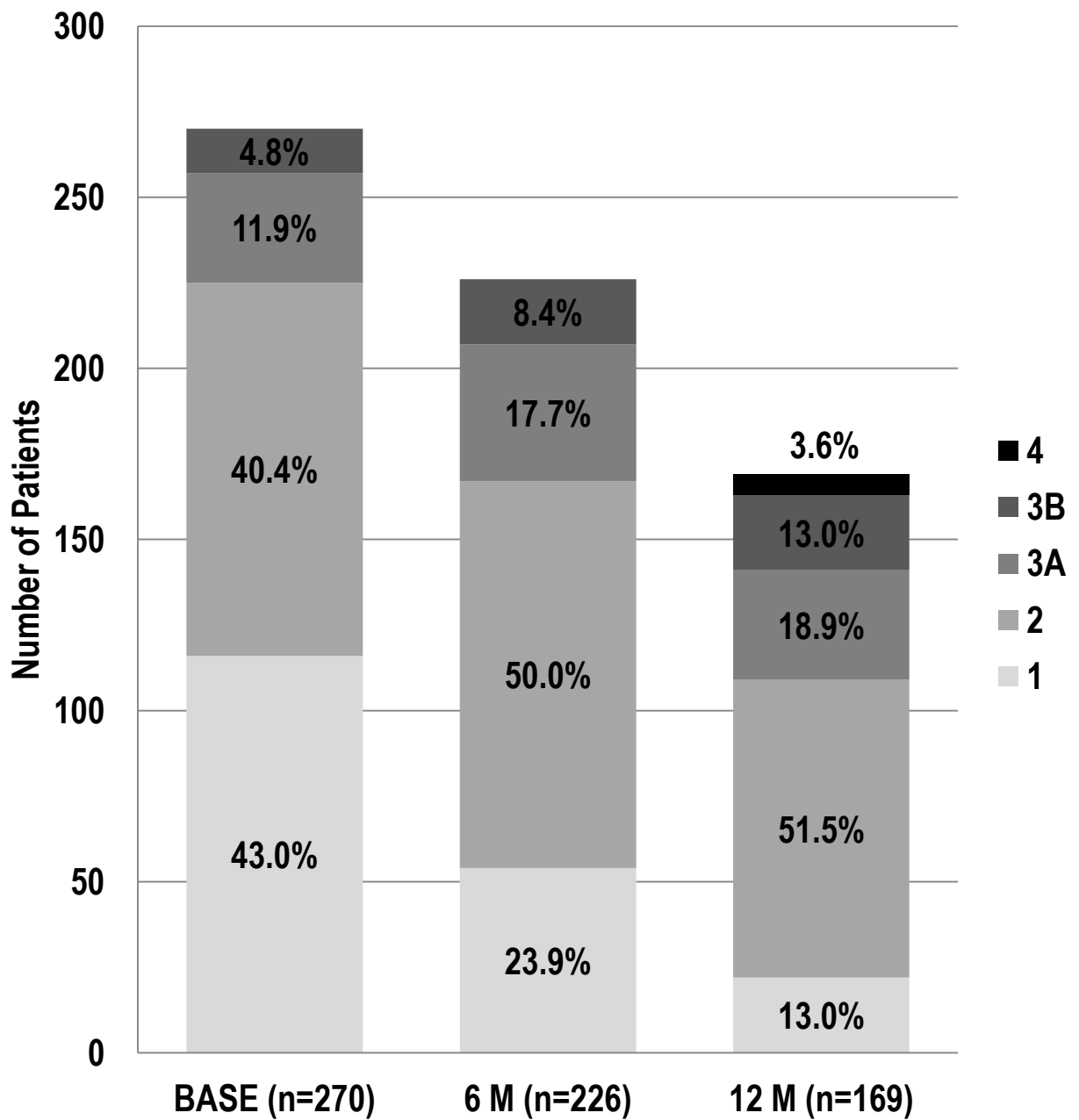

Supplement: Additional file 1 — Patient distribution according to chronic kidney disease stages. The proportion of advanced CKD stages (IIIB and IV) increased as time passed. [file 1471-2369-13-93-S1.pdf]

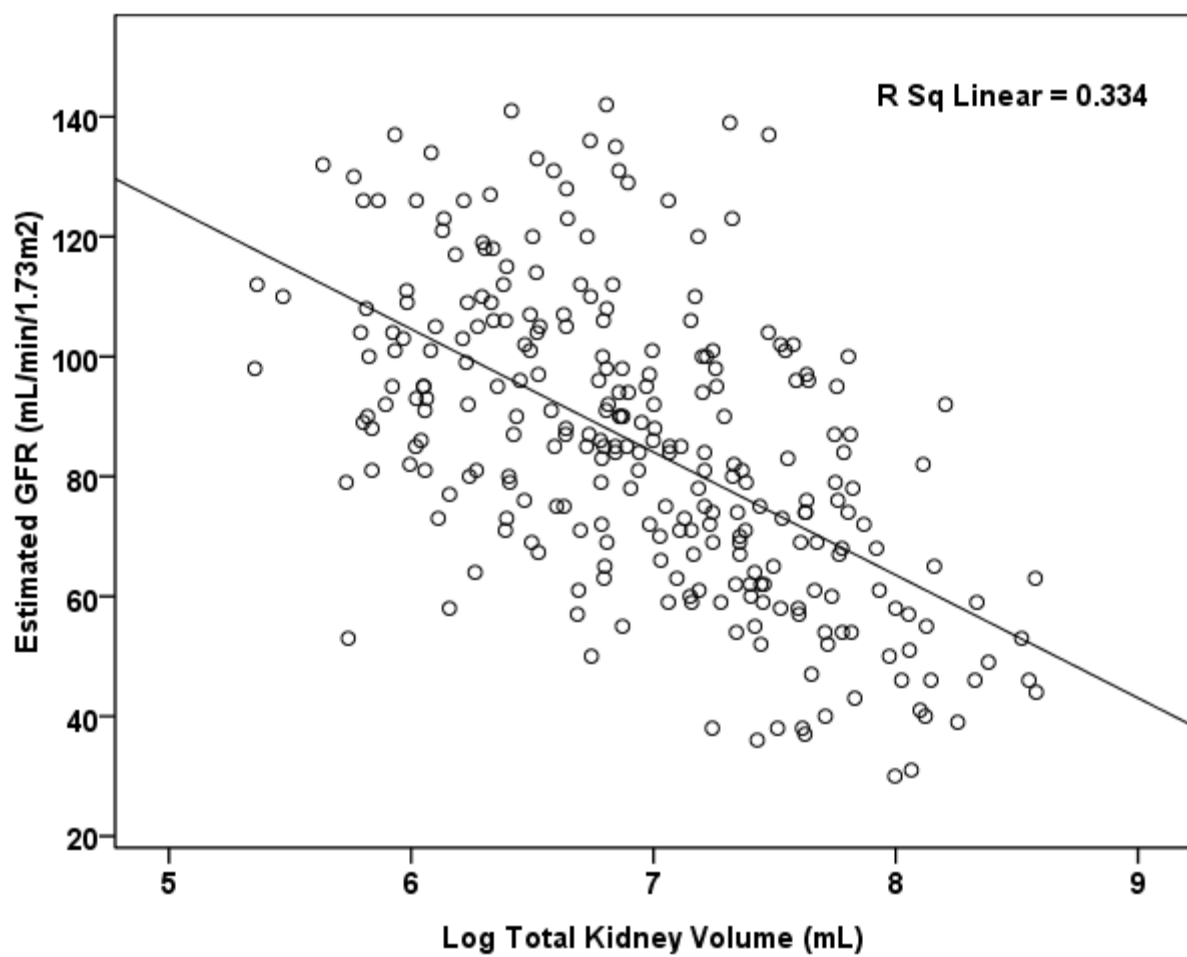

Supplement: Additional file 2 — Linear regression analysis between the estimated glomerular filtration rate and total kidney volume. The TKV was negatively correlated with the estimated GFR (r2 = 0.334, P < 0.001). [file 1471-2369-13-93-S2.pdf]

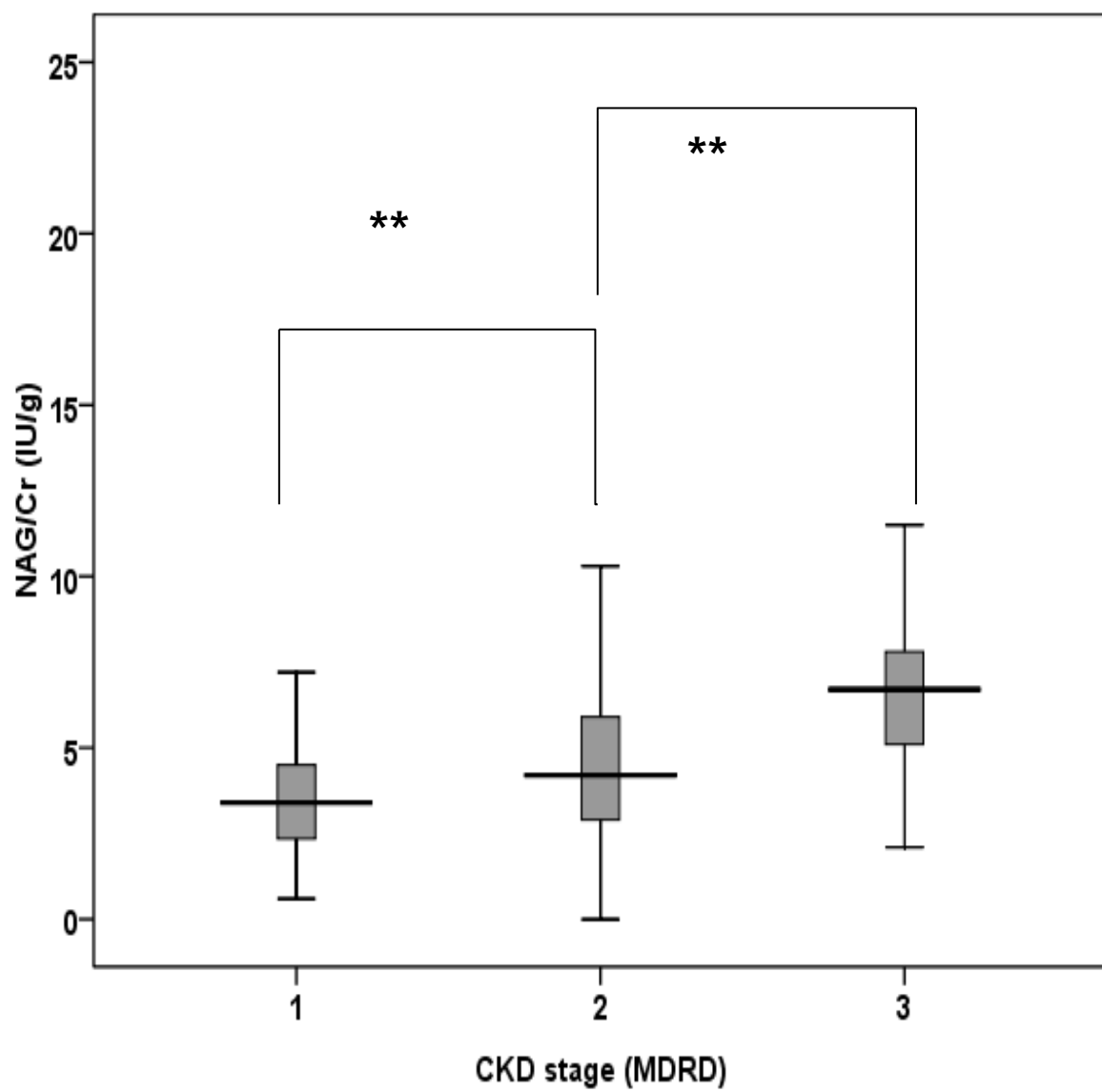

Supplement: Additional file 3 — Urinary NAG/Cr according to chronic kidney disease stages. Patients in chronic kidney disease (CKD) stage III (estimated GFR < 60 mL/min/1.73 m2) showed significantly higher urinary NAG/Cr compared with CKD stage I or II (6.48 ± 3.79 vs. 3.7 ± 2.13 vs. 4.67 ± 2.47, P < 0.001). [file 1471-2369-13-93-S3.pdf]
