# Supplementary material for: Urinary N-acetyl-β-D glucosaminidase as a surrogate marker for renal function in autosomal dominant polycystic kidney disease: 1 year prospective cohort study
Source: BMC Nephrol. 2012 Aug 30;13:93. doi: 10.1186/1471-2369-13-93 (PMC3465238; doi:10.1186/1471-2369-13-93)
Supplement: Additional file 4 — Repeated measurements of NAG/Cr could not predict renal function deterioration in 1 year. The patients were divided into three groups according to serial measurements of urinary NAG/Cr: persistently low NAG/Cr (<4.95 IU/g) (Group L-L), variable NAG/Cr (Group V), and persistently high NAG/Cr (≥4.95 IU/g) (Group H-H). Group H-H showed lower estimated GFRs at baseline (64.5 ± 23.3 mL/min/1.73 m2) and lower estimated GFR at 12 month (50.3 ± 18.5 mL/min/1.73 m2) compared to other groups (P < 0.001). Although statistically insignificant, the percentage decrement of estimated GFR over 12 months were greater in Group H-H compared to the other groups (Group H-H vs. Group V vs. Group L-L, -23.0% vs. -20.8% vs. -21.2%, P = 0.77). [file 1471-2369-13-93-S4.pdf]

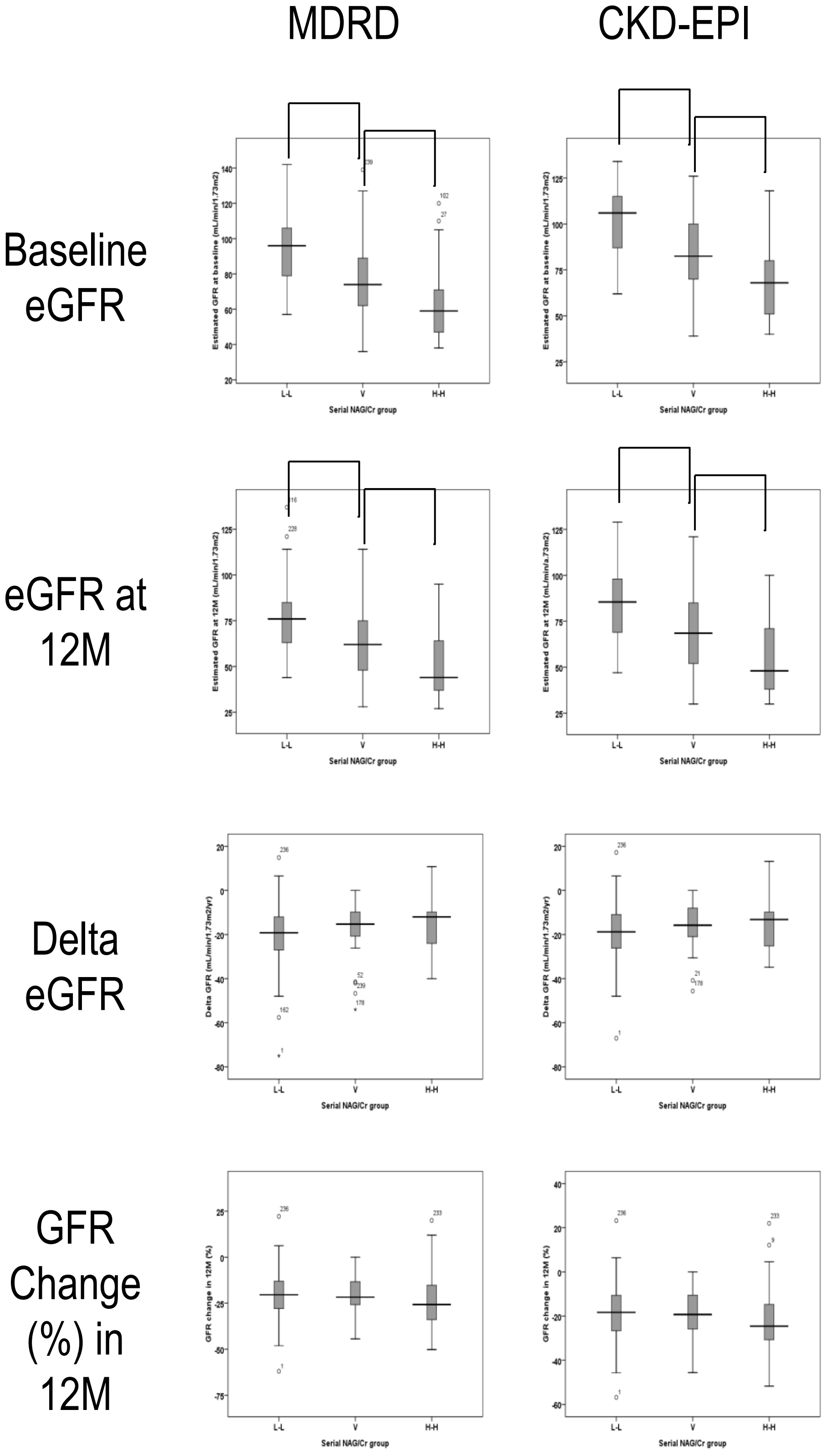

L-L: Low-Low, V: Variable, H-H: High-High
Low: NAG/Cr < 4.95, High NAG/Cr ≥ 4.95
